# Impact of Therapeutic Alcohol Administration on Perioperative Quality of Life (QoL) and Fracture Healing in Patients with Alcohol Use Disorder Undergoing Surgery for Maxillofacial Trauma—A Randomized Pilot Trial

**DOI:** 10.3390/cmtr18030037

**Published:** 2025-08-30

**Authors:** Elavenil Panneerselvam, Rajkumar Krishnan, Jaikumar Velayudham

**Affiliations:** 1 Department of Oral & Maxillofacial Surgery, SRM Dental College, SRM Institute of Science & Technology, Ramapuram Campus, Chennai 600089, India; 2 Department of Oral & Maxillofacial Pathology & Microbiology, SRM Dental College, SRM Institute of Science & Technology, Ramapuram Campus, Chennai 600089, India; dean.acad@dental.srmrmp.edu.in; 3 Department of Psychiatry, Government Medical College, Vellore 632011, India; maildrjai@rediffmail.com

**Keywords:** alcohol use disorder, maxillofacial trauma, alcohol abuse, alcohol dependence, alcohol withdrawal syndrome, perioperative care, quality of life

## Abstract

Alcohol Use Disorder (AUD) is common among patients with maxillofacial trauma. Conventional perioperative care recommends complete abstinence. However, abrupt cessation can lead to Alcohol Withdrawal Syndrome (AWS), negatively impacting psychological well-being and compliance. This randomized controlled pilot study evaluated the effectiveness of Monitored Therapeutic Alcohol Administration (MTAA) in reducing perioperative stress and enhancing quality of life without impairing fracture healing. Twenty-four adult male patients with AUD and isolated facial fractures requiring surgery were enrolled. They were assigned to either an intervention group (n = 12) receiving MTAA—oral alcohol at 0.5 g/kg/day for two weeks—or a control group (n = 12) undergoing complete abstinence. Outcomes were assessed over six weeks, including stress (Zung Self-Rating Depression Scale), quality of life (Oral Health Impact Profile-14), soft tissue healing (Landry’s Index), and hard tissue healing (Moed’s Scale, serum osteocalcin). The MTAA group showed significantly reduced stress and improved quality of life (*p* < 0.001). Healing outcomes were comparable between groups, with no significant differences in soft tissue indices, osteocalcin levels, or radiographic scores. MTAA appears to be a safe and effective strategy to manage AWS-related distress and improve postoperative recovery, offering a practical alternative to strict abstinence in the surgical management of patients with AUD.

## 1. Introduction

A significant proportion of patients (50–72%) [1,2] presenting with facial trauma have a documented history of Alcohol Use Disorder (AUD). These individuals often require surgical intervention under local or general anesthesia. Conventional management protocols dictate complete abstinence from alcohol in the perioperative period because of undesirable implications associated with alcoholism, specifically anesthetic complications, adverse drug interactions, and impaired tissue healing. However, abrupt abstinence can provoke severe emotional and psychological disturbances such as suicidal ideation, aggressive behaviour, and post-traumatic stress disorder (PTSD), which not only impact the patient but also their immediate family and the broader community. These manifestations fall under the spectrum of Alcohol Withdrawal Syndrome (AWS) [3], a condition well recognized in the current literature.

In surgical patients, alcohol abstinence can negatively influence their perception of treatment and surgical outcomes. This undesirable impact of abstinence during the perioperative period is a neglected facet. Focused research is much needed to evaluate if the benefits of alcohol abstinence truly outweigh its risks in the management of surgical patients with AUD. While various contemporary approaches exist for managing AWS, including pharmacotherapy [4], brief intervention [5], and counselling [6], their success rates are inconsistent. Some centers have adopted a harm reduction strategy termed the managed alcohol program (MAP) that involves administration of alcohol in specific quantities to manage the psychological and physiological symptoms of alcohol withdrawal [7]. This approach aims to reduce the adverse consequences of abstinence, such as the consumption of non-beverage/surrogate alcohol (sanitizers/paint thinners), withdrawal seizures, police encounters, and emergency hospitalizations. Additionally, non-compliance with treatment and discharge against medical advice are common.

Gradual alcohol reduction protocols implemented before surgery have shown promise when sufficient preoperative time is available to condition the patient [8]. However, in trauma scenarios that require urgent surgical intervention, such conditioning is often not feasible. Despite the clinical relevance, there is limited literature addressing the management of maxillofacial trauma in patients with AUD, especially regarding perioperative alcohol protocols. Studies that assess both localized surgical outcomes and broader psychosocial recovery are particularly scarce.

The purpose of this study was to evaluate the potential benefits of monitored therapeutic alcohol administration (MTAA) in improving perioperative quality of life and fracture healing among patients with AUD undergoing surgery for maxillofacial trauma. The specific objectives included comparison of perioperative stress, quality of life, and fracture healing in alcohol-dependent patients undergoing complete abstinence versus those managed with MTAA, using clinical and biochemical parameters.

## 2. Materials and Methods

The present study included patients who presented to the Department of Oral and Maxillofacial Surgery for treatment of mandibular fractures. The study protocol was explained to the patients and family members, who provided written informed consent for participation in the study (Supplementary Material S2). The patients’ demographic details, including age, gender, marital status, ethnicity, language use, geographic and socio-economic parameters (e.g., rural/urban location, employment status, income, education) were collected. The type of alcohol, quantity, and duration of habit were also documented.

The following criteria were considered for patient selection:

1. Adults aged 25–50 years, taking into consideration the official age in India for alcohol consumption (21 years) and the age range with peak bone mass in the adult skeleton (20–50 years) [9]
2. Diagnosed with AUD—mild and moderate grade (DSM-5 criteria) [10]
3. AUDIT scale—risk zone 1, 2, and 3 (Alcohol Use Disorder Identification Test by WHO) [11]
4. CIWA-Ar score < 14 (Clinical Institute Withdrawal Assessment of Alcohol Scale—Revised) [12]
5. Daily intake of at least 60 g of Ethanol (150 mL of distilled spirit) for the last 1 year
6. ASA Grade III (due to alcoholism)
7. GCS score of 15 and ambulatory and
8. Diagnosed with isolated mandibular fractures requiring surgical intervention.

Exclusion criteria considered were:

1. Patients less than 21 years and greater than 50 years
2. Patients with head injury and polytrauma
3. Patients with systemic problems that compromise wound healing
4. Patients with “drinking in moderation” (2 drinks or less in a day for men; 1 drink or less for women, according to the National Institute on Alcohol Abuse and Alcoholism)
5. Patients with smoking
6. Patients who are non-compliant with the perioperative protocol
7. AUDIT zone 4, CIWA-Ar score > 14
8. AST/ALT ratio more than 2 (AST—aspartate aminotransferase; ALT—alanine aminotransferase)
9. Patients not consenting to enrolment into the study

The institutional ethics committee approved the present study (SRMDC/IRB/2022/PhD/No.151) (Supplementary Material S5), which was conducted in accordance with the Consolidated Standards of Reporting Trials statement (Supplementary Material S3 and S4) and the criteria of the Declaration of Helsinki, the ethical principles for medical research involving human participants.

### 2.1. Study Design

This was a single-blinded, randomized, controlled clinical trial involving twenty-four patients with a history of alcohol dependence, enrolled and randomized into two groups (n = 12 each) using computer-generated random allocation. The study group received MTAA during the perioperative period, while the control group underwent complete abstinence from alcohol. The outcome assessors (Zung’s, Oral Health Impact Profile (OHIP) score, Landry and Moed scores) were blinded to the intervention.

### 2.2. MTAA Protocol

The study group received oral intake of a standardized alcoholic beverage, rum, at a dose of 0.5 g/kg/day (1.4 mL/kg/day) [13] for a duration of 7 days, followed by administration of 0.7 mL/kg/day for the second week, administered daily. The patient was recalled as an outpatient for alcohol administration every day, one hour after breakfast. This was supervised and monitored by a member of the investigating team using a calibrated digital breath alcohol analyzer twice daily (one hour and 12 h after alcohol administration) to ensure compliance. The device threshold for the breath analyzer we used was 0.03% and the acceptable deviation was set at 10% of the Blood Alcohol Concentration (BAC) value (0.01% BAC) [14]. The control group abstained completely from alcohol during the study period.

### 2.3. Surgical Technique and Postoperative Management

All patients were operated on by the same maxillofacial surgeon (E.P.) using standardized techniques appropriate to the fracture type. A vestibular and retromandibular incision was used to expose the fracture for non-condylar fractures and condyle fractures, respectively. Fixation was performed as indicated by the fracture line and in accordance with Champy’s and Meyer’s lines of osteosynthesis; the body fracture underwent fixation with 1 miniplate, the symphysis and para-symphysis fracture with 2 miniplates, and condyle fractures with two miniplates. Closure of the fracture site was performed in layers.

Postoperative medications included intravenous Cefotaxime (1 g) followed by oral Cefixime (200 mg) for prevention of infection, intravenous diclofenac (75 mg) followed by oral diclofenac (50 mg) for pain management, and intravenous Dexamethasone (8 mg) for anti-inflammatory effect. All patients were discharged on the second postoperative day and were called for reviews on an outpatient basis.

The patients were managed by an interdisciplinary team including a maxillofacial surgeon, an anesthetist, and a psychiatrist. Also, careful monitoring for any symptoms of AWS was performed for 2 months, along with periodic counselling to minimize alcohol consumption.

### 2.4. Patient Safety Protocol

A protocol was in place to manage patients if they required medication/treatment for management of AWS during the study period in accordance with the CIWA-Ar criteria [12,15]. Adverse events, protocol violations, AWS episodes, and emergency interventions were documented, and these patients were managed with the prompt institution of monitoring and treatment by the psychiatrist.

Criteria for discontinuation from the study included (i) Patients who did not adhere to the protocol for the study period, (ii) patients who required treatment for AWS episodes or emergency medical interventions, and (iii) voluntary withdrawal of consent.

All patients were provided psychiatric counselling during the study period and were made aware of anonymous, self-supporting fellowship programs such as Alcoholics Anonymous, to facilitate complete abstinence in the long run and the benefits of enrolling in them.

### 2.5. Outcome Measures

Outcomes were evaluated using clinical, radiographic, and biochemical parameters.

#### 2.5.1. Perioperative Stress

Zung’s Self-Rating Depression Scale [16] (SDS) was used to assess stress at five intervals: post-trauma (baseline), postoperative day 2, postoperative week 1, week 2, week 4, and week 6. SDS scores were interpreted as: 25–49: normal, 50–59: mild depression, 60–69: moderate depression, and ≥70: severe depression.

#### 2.5.2. Quality of Life

The Oral Health Impact Profile-14 [17] (OHIP-14) questionnaire assessed functional and psychosocial well-being across seven domains at the same time points. Total scores ranged from 0 (excellent) to 56 (poor), with thresholds defined as 0–14: excellent, 15–28: good, 29–42: bad, and 43–56: poor.

#### 2.5.3. Soft Tissue Healing

Soft tissue healing was evaluated using the Modified Landry’s Wound healing index [18] on postoperative days 5, 9, 15, and 21. Healing scores ranged from 1 (very poor) to 5 (excellent), based on tissue colour, bleeding, epithelialization, and granulation.

#### 2.5.4. Hard Tissue Healing

Hard tissue healing was evaluated through both biochemical and radiographic parameters:

- Serum osteocalcin levels were measured via ELISA preoperatively and on postoperative day 30.
- Radiographic assessment was performed using orthopantomograms at the preoperative phase, 4th and 8th weeks postoperatively, and graded using Moed’s Scale [19]: Score 1: No callus formation, 2: Minimal callus, 3: Moderate callus, and 4: Complete callus bridging.

A reporting of safety outcomes was also performed.

### 2.6. Statistical Analysis

The Normality tests by Kolmogorov–Smirnov and Shapiro–Wilks reveal that all variables except S. Osteocalcin (Biochemical) follow normal distribution. Therefore, to analyze the data, both parametric and non-parametric methods were applied. For variables that follow a normal distribution, the independent samples *t*-test was applied to compare means between the study and control groups, while the paired *t*-test was applied to compare means between the first time point and subsequent time points. The independent samples Mann–Whitney U test was applied to compare means for S. Osteocalcin (Biochemical) exclusively, which did not follow normal distribution. To compare values between pre-op and post-op time points, the Wilcoxon Signed Rank test was used. For all mean values, mean differences, and median values, 95% confidence limits were calculated. To analyze the data, SPSS (IBM SPSS Statistics for Windows, Version 26.0, Armonk, NY, USA: IBM Corp. Released 2019) was used, and the significance level was fixed at 5% (α = 0.05).

## 3. Results

The study included 24 participants, with 12 each in the study and control groups. The mean age was comparable between the study (36.0 ± 6.2 years) and control (34.8 ± 7.1 years) groups, with no statistically significant difference (*p* = 0.428). The data analyzed indicated significant differences between the study and control groups across various parameters, including serum osteocalcin levels, Moed scores, Landry scores, Zung’s stress scores, and OHIP quality of life (QoL) scores (Table 1) (Supplementary Material S1).

### 3.1. Stress Levels (Zung Score)

**Figure 1:**
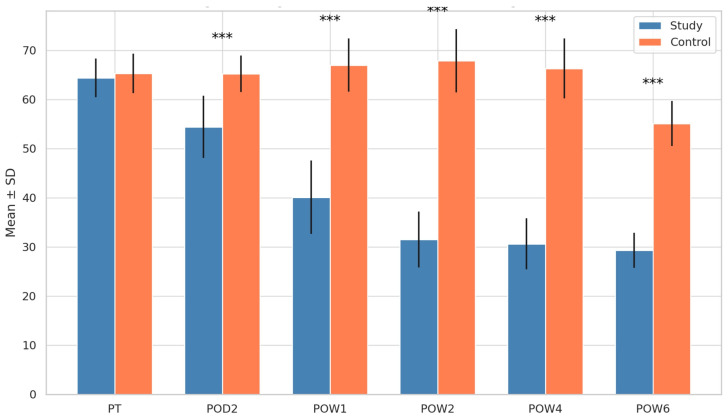
Comparison of Zung’s score for stress between groups. *** = *p* < 0.001.

Both groups had comparable baseline (post-trauma) Zung scores (*p* = 0.577). From POD2 through POW6, the study group showed a consistent and statistically significant reduction in stress scores (*p* < 0.001 at all time points). In contrast, the control group maintained elevated stress levels until POW4, with a significant reduction observed only at POW6 (*p* < 0.001). Intergroup comparisons showed significantly lower stress scores in the study group from POD2 onward (*p* < 0.001 at all time points) (Figure 1).

### 3.2. Quality of Life (OHIP Score)

**Figure 2:**
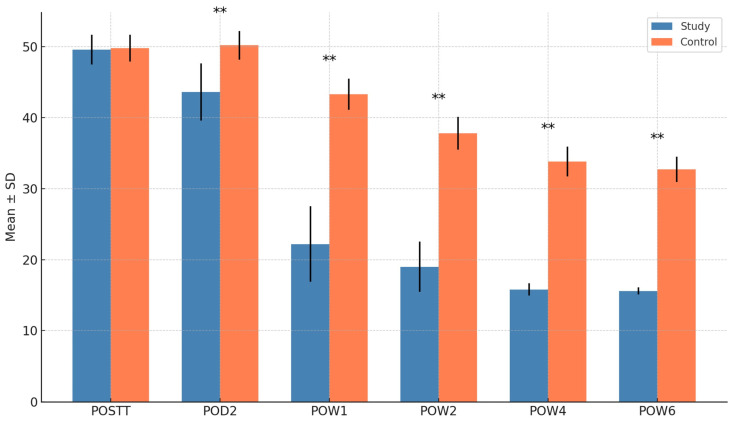
Comparison of OHIP score for quality of life between groups. ** = *p* < 0.05.

Baseline OHIP scores were similar between groups (*p* = 0.763). The study group demonstrated a significant improvement in quality of life from POD2 to POW6 (*p* < 0.001 at all time points). While the control group also showed improvements, the magnitude was significantly lower. Between-group comparisons revealed significantly lower OHIP scores (indicating better quality of life) in the study group at all postoperative time points from POD2 onward (*p* < 0.001) (Figure 2).

### 3.3. Soft Tissue Healing (Landry Score)

**Figure 3:**
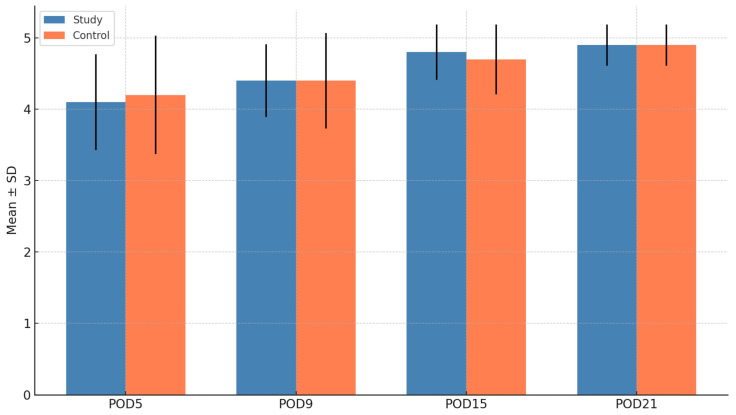
Comparison of the Landry score for bone healing between groups.

Landry scores improved over time in both groups. Intergroup comparisons showed no statistically significant differences at POD5, POD9, POD15, or POD21 (all *p* > 0.05) (Figure 3). Within the study group, statistically significant improvements were noted between POD5 and later time points (POD9: *p* = 0.039; POD15: *p* = 0.002; POD21: *p* < 0.001). Similar trends were observed in the control group between POD5 and POD15 (*p* = 0.007) and POD21 (*p* = 0.005).

### 3.4. Biochemical Marker (Serum Osteocalcin)

**Figure 4:**
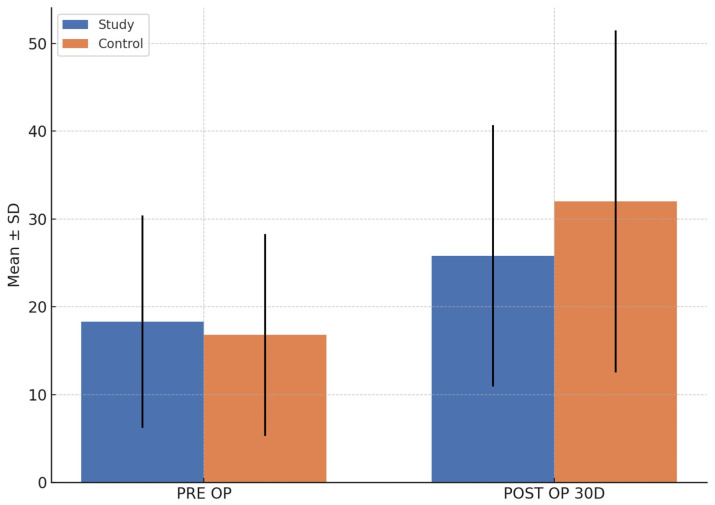
Comparison of serum osteocalcin levels between groups.

Despite there being perceptible differences between the groups for the pre- and postoperative means, they were not statistically significant (pre-op: *p* = 0.319; post-op: *p* = 0.478) (Figure 4). However, within-group comparisons revealed significant postoperative increases in both the study (*p* = 0.003) and control (*p* = 0.002) groups.

### 3.5. Hard Tissue Healing (Moed Score)

**Figure 5:**
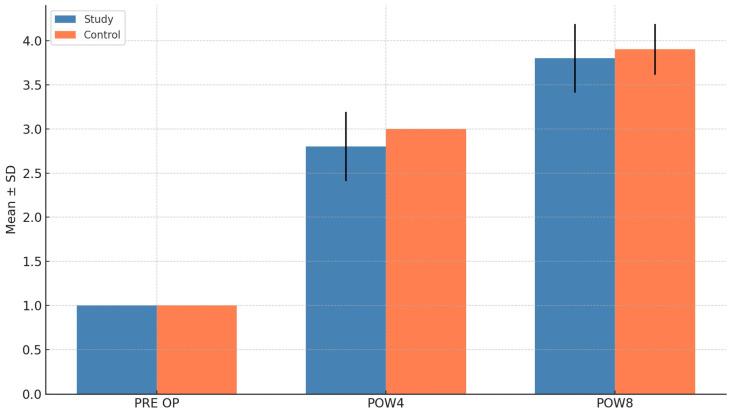
Comparison of Moed score for bone healing between groups.

Both groups showed progressive improvement in Moed scores from baseline (preoperative) through postoperative weeks 4 and 8. There was no statistically significant difference between the study and control groups at POW4 (*p* = 0.166) and POW8 (*p* = 0.557). Within-group analysis demonstrated significant improvement in both groups from baseline to POW4 and POW8 (*p* < 0.001) (Figure 5).

### 3.6. Safety Outcomes

A comprehensive safety assessment was conducted throughout the study period. All patients were monitored closely for adverse events, protocol violations, signs of alcohol withdrawal, need for emergency interventions, and adherence to discontinuation criteria.

#### 3.6.1. Adverse Events

There were no adverse events recorded during this study involving 24 subjects (0%). Most patients had mild symptoms such as sweating and insomnia, while none demonstrated any moderate or severe symptoms. There were no patients needing emergency interventions.

#### 3.6.2. Protocol Violations

There were no protocol violations in this pilot group.

#### 3.6.3. Alcohol Withdrawal Syndrome (AWS) Episodes

- Prevalence: Most patients exhibited mild symptoms as discussed, with none presenting moderate or severe symptoms.
- Management: There were no patients requiring pharmacological help or admission. The patients were treated by the psychiatrist with examination and counselling. No cases of delirium tremens or seizures were reported.

All patients recovered without complications and completed follow-up.

## 4. Discussion

The present study aimed to evaluate the efficacy of MTAA in improving perioperative quality of life and its impact on fracture healing in AUD patients undergoing surgery for maxillofacial trauma. The findings revealed significant differences between the MTAA and abstinence groups in terms of perioperative stress and quality of life, while no significant differences were observed in hard or soft tissue healing. These results contribute to the growing body of literature on the perioperative management of alcohol-dependent patients and highlight the potential benefits of MTAA in mitigating psychological distress during recovery.

### 4.1. Perioperative Stress and Quality of Life

It is well-documented that AWS exacerbates perioperative stress, leading to agitation, depression, and even delirium tremens in severe cases [3,20]. The abrupt cessation of alcohol in dependent individuals induces neurochemical imbalances, particularly within the GABAergic and glutamatergic systems, exacerbating anxiety and depressive symptoms [21], which often result in non-compliance with postoperative care and hospitalization [13].

The findings of the present study align with studies demonstrating that abstinence contributes to poor quality of life, reduced emotional well-being, and decreased patient-perceived recovery. The MTAA group’s lower stress scores at all postoperative intervals suggest that controlled alcohol administration minimizes withdrawal-induced psychological distress, thereby improving patient cooperation and overall satisfaction. This is particularly critical in patients with maxillofacial trauma, whose psychological well-being is already compromised due to alterations in facial esthetics and essential functions such as mastication and speech [16]. These factors amplify the stress associated with alcohol abstinence, thereby adversely impacting functional recovery and adherence to rehabilitation protocols [17].

### 4.2. Tissue Healing Outcomes

Contrary to conventional concerns regarding tissue healing in alcoholism, the study found no significant differences in hard or soft tissue healing between the MTAA and abstinence groups. Both groups exhibited satisfactory fracture healing with comparable improvements in serum osteocalcin levels, radiographic bone healing, and soft tissue recovery. However, this finding contrasts with studies highlighting alcohol’s detrimental effects on wound healing [13]. The discrepancy may be attributed to the controlled, moderate dosing in the MTAA protocol, which potentially avoids the toxic thresholds associated with heavy alcohol use. This property of alcohol eliciting physiologically safe outcomes when administered in small doses is referred to as hormetic behaviour [22,23,24]. In the present study, measures were taken to enable standardization of healing potential following fracture as well as comparison of outcomes; the inclusion criteria consisted of subjects (i) within the age range of 25 to 50, in whom the bone mass was stable, contrary to patients above 65, in whom bone healing is highly compromised [9,25] and (ii) with mandibular fractures, not involving any other facial bones to negate differential healing potential between facial bones. However, further research is needed to explore dose-dependent effects and long-term healing outcomes.

### 4.3. Evolution of Alcohol Therapy

The administration of alcohol to manage AWS has remained a debatable concept. Its use has been criticized by many because of its low therapeutic index, gastric irritation, and potential systemic complications [3,26,27]. However, proponents of ethanol therapy specifically from surgical/trauma units justify its effective usage in AWS, specifically in current active abuse [28], patients at high risk of AWS, previous history of withdrawal [20], in scenarios where benzodiazepine is ineffective, or situations with inadequate time for detoxification [29]. Alcohol relieves the symptoms of AWS without the excessive sedation and respiratory depression associated with Benzodiazepines, which are considered the first-line drug for AWS [21,22,28]. Moreover, in contrast to benzodiazepines, low concentrations of ethanol have been demonstrated to exert immunostimulatory effects by modulating the hypothalamic–pituitary–adrenal (HPA) axis, specifically through the suppression of cortisol secretion, thereby attenuating the deleterious physiological consequences of stress-induced hypercortisolaemia [13,30] (Table 2).

The concept of MTAA in this study parallels the principles of Managed Alcohol Programs (MAPs), which are structured harm-reduction interventions designed to provide regulated alcohol doses to dependent individuals in clinical settings [7]. MAPs have been successfully implemented in hospitals and long-term care facilities to stabilize patients with severe alcohol use disorder (AUD), reducing withdrawal complications, emergency room visits, and self-destructive behaviors [35,36]. Literature reports the administration of alcohol via intravenous (5%/10% ethanol), oral, and nasogastric (Ryle’s) routes, based on clinical needs. Critics argue that providing alcohol in healthcare settings may enable addiction, but evidence suggests that MAPs do not increase long-term alcohol consumption and may facilitate eventual AUD treatment [37]. Hence, many institutional protocols recommend ethanol therapy and even permit official alcohol prescription by doctors, delivery by formularies, and administration by healthcare professionals [38]. The ethical justification lies in prioritizing immediate patient safety and surgical recovery, as withdrawal poses a greater acute risk than controlled alcohol use [4]. The integration of MTAA or MAP-like protocols in perioperative care represents a pragmatic harm-reduction strategy for alcohol-dependent surgical patients and beneficial in many ways; reduction in AWS complications such as of delirium, seizures, and cardiovascular stress [28], improved engagement with care and adherence to postoperative instructions when withdrawal symptoms are minimized [39], and lowering burden on healthcare systems by reducing ICU admissions and hospital stays [13]. By addressing withdrawal-related stress and improving quality of life, these approaches complement traditional surgical management without compromising tissue healing. As healthcare moves toward patient-centred models, structured alcohol management should be considered a viable option for high-risk AUD populations undergoing trauma surgery.

Key similarities between MTAA and MAPs include (i) supervised dosing: Both protocols administer alcohol in controlled, measured quantities to prevent intoxication while avoiding withdrawal [23], (ii) biochemical monitoring using breathalyzer or blood alcohol concentration (BAC) tracking to ensure compliance and safety [40] and (iii) multidisciplinary integration between surgeons, psychiatrists, and addiction specialists to optimize outcomes [41]. The present study, however, differs from current literature in several key aspects. It offers substantive evidence indicating that osseous fracture healing, as well as adjacent soft tissue recovery, are not adversely affected by low-dose alcohol administration. Notably, beginning on the third postoperative day, patients were not confined to hospital or community-based care settings but were permitted to resume their routine activities. This approach significantly enhanced the quality of life in the MTAA group. Patients were monitored over a two-month follow-up period, corresponding to the typical duration of maxillofacial fracture healing. Importantly, there were no losses to follow-up, suggesting a high level of patient adherence, likely influenced by improved psychological well-being. The oral administration of alcohol, especially in a tapered fashion, appeared to provide psychological comfort, which may have contributed to enhanced mood and motivation to reduce or stop the consumption of alcohol in the future [13]. In addition, consumption of alcohol closer to the mealtime reduced the ill effects of alcohol. Delivering alcohol in a controlled, supervised clinical environment also fostered a sense of safety and reassurance among both patients and their families. This supportive context may contribute to an earlier return to occupational activity in the study group compared to controls. The significant positives of the study include the specific number of days of oral administration, which corresponds to the possible peaking and remission of the severe form of AWS, delirium tremens. This ensured complete physiological and psychological recovery and stability of the patient [4]. The study also prescribed only medication (antibiotics, analgesics, and steroids), which did not have any potential for interaction with alcohol in the perioperative period [42]. None of the patients required rehospitalization due to AWS.

### 4.4. Clinical Implications and Ethical Considerations

The study supports the growing interest in harm-reduction strategies for alcohol-dependent surgical patients and improving patient compliance in hospital settings [28,35,36,41]. However, ethical and logistical challenges, such as ensuring strict supervision to prevent misuse and addressing societal stigma, must be carefully navigated [4,39]. Though institutional approval and monitored administration in this study provide a framework for safe implementation, broader clinical adoption requires standardized protocols and multidisciplinary collaboration.

### 4.5. Limitations and Future Directions

The study’s small sample size and short follow-up period limit the generalizability of the findings. Additionally, the predominance of male participants may introduce gender bias, as alcohol dependence and withdrawal manifestations can differ by sex [43]. To address these limitations, a clinical trial based on this pilot study is underway, including larger, more diverse cohorts (patients stratified based on involvement of facial bones, pharmacological regimens, AUDIT, and CIWA-Ar scores) [11,12] and longer follow-ups. While this study supports MTAA’s psychological benefits, further research should (i) compare MTAA with pharmacological AWS treatments (e.g., benzodiazepines) in surgical cohorts, (ii) evaluate long-term outcomes, including relapse rates beyond 8 weeks, (iii) assess cost-effectiveness and scalability in diverse healthcare settings, and (iv) use of CDT (carbohydrate deficient transferrin) assay which is a more sensitive and specific method to monitor drinking patterns in the follow-up period, over a longer duration [44].

## 5. Conclusions

This study suggests that MTAA can significantly reduce perioperative stress and enhance quality of life in alcohol-dependent patients without adversely affecting tissue healing. While the physiological outcomes were comparable between groups, the psychological benefits of MTAA underscore its potential as a complementary approach in surgical management. These findings contribute to the evolving concepts of harm reduction in perioperative care and advocate for patient-centered strategies in managing alcohol dependence.

## Figures and Tables

**Table 1:** Summary of outcome variables.

**N = 12**	**Groups**						
	**Study**			**Control**			
**Gender**	**Frequency**						
Female	0			0			
Male	12			12			
**Age (Years)**	**Mean (S.D)**			**Mean**			***p* value**
	36 (6.2)			34.8 (7.1)			0.428
**Zung Score (Stress)**	**Mean (S.D)**	**95% CI**		**Mean**	**95% CI**		***p* value**
		**LL**	**UL**		**LL**	**UL**	
	64.4 (3.92)	61.9	66.9	65.3 (4.01)	62.8	67.9	0.577
	54.4 (6.33)	50.4	58.4	65.2 (3.71)	62.8	67.5	<0.001 ***
	40.1 (7.49)	35.3	44.8	67 (5.44)	63.5	70.5	<0.001 ***
	31.5 (5.68)	27.9	35.1	67.8 (6.44)	63.8	72.0	<0.001 ***
	30.6 (5.20)	27.3	33.9	66.3 (6.10)	62.5	70.2	<0.001 ***
	29.3 (3.55)	27.1	31.6	55.1 (4.56)	52.2	58.0	<0.001 ***
**OHIP Score** **(QoL)**	**Mean (S.D)**	**LL**	**UL**	**Mean**	**LL**	**UL**	***p* value**
	49.6 (2.11)	48.2	50.9	49.8 (1.90)	48.6	51.0	0.763
	43.6 (4.03)	41.0	46.1	50.2 (2.04)	48.9	51.5	<0.001 ***
	22.2 (5.31)	18.8	25.5	43.3 (2.18)	41.9	44.6	<0.001 ***
	19 (3.54)	16.7	21.3	37.8 (2.29)	36.4	39.3	<0.001 ***
	15.8 (0.87)	15.2	16.3	33.8 (2.09)	32.4	35.1	<0.001 ***
	15.6 (0.51)	15.3	15.9	32.7 (1.78)	31.5	33.8	<0.001 ***
**Landry Score (Soft tissue)**	**Mean (S.D)**	**LL**	**UL**	**Mean**	**LL**	**UL**	***p* value**
	4.1 (0.67)	3.7	4.5	4.2 (0.83)	3.6	4.7	0.790
	4.4 (0.51)	4.1	4.7	4.4 (0.67)	4.0	4.8	0.999
	4.8 (0.39)	4.6	5.1	4.7 (0.49)	4.4	5.0	0.368
	4.9 (0.29)	4.7	5.1	4.9 (0.29)	4.7	5.1	0.999
**Moed Score** **(Hard tissue)**	**Mean (S.D)**	**LL**	**UL**	**Mean**	**LL**	**UL**	***p* value**
	1 (0)	1.0	1.0	1 (0)	1.0	1.0	-
	2.8 (0.39)	2.6	3.1	3 (0)	3.0	3.0	0.166
	3.8 (0.39)	3.6	4.1	3.9 (0.29)	3.7	4.1	0.557
**S. Osteocalcin** **(Biochemical)**	**Median**	**LL**	**UL**	**Median**	**LL**	**UL**	***p* value**
	11.6	4.7	22.8	18.1	8.9	28.2	0.319
	22.6	12.5	30.0	33.1	14.0	48.7	0.478

*** = *p* < 0.001.

**Table 2:** Benzodiazepines vs. Harm Reduction Strategies in AWS Management [12,15,31,32,33,34].

	Benzodiazepines (Pharmacological Management)	Harm Reduction Strategies
**Primary Role**	Mainstay treatment for acute AWS	Used in many surgical ICUs to treat AWS A broader approach to reducing the harms of AWS and long-term alcohol use
**Mechanism**	Enhances GABAergic neurotransmission to dampen CNS hyperexcitability during alcohol cessation	Immunostimulatory effect by modulating the hypothalamic–pituitary–adrenal axis and suppression of cortisol. Uses medical, psychosocial, and supportive measures to mitigate risks without requiring immediate abstinence
**Regimens**	Fixed dose Symptom-triggered protocols	Flexible, individualized approaches based on patient readiness and context
**Main Advantages**	High efficacy in preventing seizures and delirium tremors Rapid stabilization	Safer for long-term use; addresses psychosocial determinants Adaptable for those unwilling/unable to stop drinking immediately
**Limitations/Risks**	Risk of dependence, cross-tolerance, sedation, and respiratory depression	May not rapidly control severe withdrawal; requires longer engagement and multidisciplinary support
**Ideal indications**	Acute inpatient management of moderate-to-severe AWS	Long-term harm mitigation, resource-limited settings, trauma cases/when abstinence is not immediately achievable
**Outcome Focus**	Immediate symptom relief Complication prevention	Sustainable reduction in alcohol-related harm and Improved quality of life

## Data Availability

The data for the study is available on request.

## Supplementary Materials

The following supporting information can be downloaded at: https://www.mdpi.com/article/10.3390/cmtr18030037/s1. S1. Raw data in Excel format, S2. Blank copy of informed consent form used for the study, S3. Copy of the CONSORT flowchart, S4. Copy of the CONSORT checklist, S5. Registration details with Clinical trials registry—India.—CTRI/2025/03/082706 dated 19 March 2025. Reference [45] is cited in the Supplementary Materials.
