# Supplementary material for: Impact of Therapeutic Alcohol Administration on Perioperative Quality of Life (QoL) and Fracture Healing in Patients with Alcohol Use Disorder Undergoing Surgery for Maxillofacial Trauma—A Randomized Pilot Trial
Source: Craniomaxillofac Trauma Reconstr. 2025 Aug 30;18(3):37. doi: 10.3390/cmtr18030037 (PMC12452396; doi:10.3390/cmtr18030037)
Supplement: Supplementary file 1 [file cmtr-18-00037-s001.zip › 2. Patient consent form English.pdf]

SRM DENTAL COLLEGE

RAMAPURAM, CHENNAI-89

CONSENT FORM

I, _____________, hereby give full consent to Dr. Elavenil Panneerselvam of SRM Dental College, Ramapuram, to perform the procedure _________________________________ on me. I have been explained in detail about the procedure, medications, and all possible complications that may arise due to it.

For educational purposes, pre- and post-operative photos and videos may be taken and published.

I understand that I must undergo certain investigations to diagnose and treat my condition. My doctor has explained the purpose of this research/study to me in a language that I understand.

I wholeheartedly agree for the doctor to use the results of the treatment for medical research activities, provided my personal identity is not revealed to anyone. The institution or doctor will not be held responsible for any unforeseen situations.

I understand that I can withdraw from this research/study at any time, and it will not affect the quality of healthcare or treatment given to me.

I also confirm that I am able to read and write in the English language.

Signature / Left Thumb Impression of the Patient

Signature of the Surgeon/Investigator

Signature / Left Thumb Impression of the Attendant
