# Supplementary material for: Impact of Therapeutic Alcohol Administration on Perioperative Quality of Life (QoL) and Fracture Healing in Patients with Alcohol Use Disorder Undergoing Surgery for Maxillofacial Trauma—A Randomized Pilot Trial
Source: Craniomaxillofac Trauma Reconstr. 2025 Aug 30;18(3):37. doi: 10.3390/cmtr18030037 (PMC12452396; doi:10.3390/cmtr18030037)

Dr Anant Results 01-08-2025

(If P-Value is <0.05 then statistically significant)

The Normality tests Kolmogorov-Smirnov and Shapiro-Wilks tests results reveal that all variables except S. Osteocalcin (Biochemical) follow Normal distribution. Therefore, to analyse the data, both parametric and non-parametric methods are applied.

For variables that follow a Normal distribution, to compare mean values between the Study and Control groups, an independent samples t-test is applied. To compare mean values between the first time-point and subsequent time points paired t-test is applied.

The variable S. Osteocalcin (Biochemical), which do not follow a Normal distribution, to compare values between the Study and Control Groups, the independent samples Mann-Whitney U test is applied. To compare values between pre-op and post-op time points, the Wilcoxon Signed Rank test is applied.

For all mean values, mean differences and median values, 95% confidence limits are calculated.

To analyse the data SPSS (IBM SPSS Statistics for Windows, Version 26.0, Armonk, NY: IBM Corp. Released 2019) is used. The significance level is fixed at 5% (α = 0.05).

Independent samples T-Test to compare mean age between Study and Control Groups

	Group	N	Mean	SD	p-value
Age (years)	Study	12	36.0	6.223	0.428
	Control	12	34.8	7.095	

Descriptive Statistics and independent samples t-test to compare mean values between Study and Control Groups

Moed score (Hard tissue)	Group										p-val
	Study					Control					
	N	Mean	SD	LL	UL	N	Mean	SD	LL	UL	
Pre-op	12	1.0	.00	1.0	1.0	12	1.0	.00	1.0	1.0	-
POW4	12	2.8	.39	2.6	3.1	12	3.0	.00	3.0	3.0	0.166
POW8	12	3.8	.39	3.6	4.1	12	3.9	.29	3.7	4.1	0.557

Landry score (Soft tissue)	Group										p-val
	Study					Control					
	N	Mean	SD	LL	UL	N	Mean	SD	LL	UL	
POD5	12	4.1	.67	3.7	4.5	12	4.2	.83	3.6	4.7	0.790
POD9	12	4.4	.51	4.1	4.7	12	4.4	.67	4.0	4.8	0.999
POD15	12	4.8	.39	4.6	5.1	12	4.7	.49	4.4	5.0	0.368
POD21	12	4.9	.29	4.7	5.1	12	4.9	.29	4.7	5.1	0.999

Zung score (Stress)	Group										p-val
	Study					Control					
	N	Mean	SD	LL	UL	N	Mean	SD	LL	UL	
Post-trauma	12	64.4	3.92	61.9	66.9	12	65.3	4.01	62.8	67.9	0.577
POD2	12	54.4	6.33	50.4	58.4	12	65.2	3.71	62.8	67.5	<0.001
POW1	12	40.1	7.49	35.3	44.8	12	67.0	5.44	63.5	70.5	<0.001
POW2	12	31.5	5.68	27.9	35.1	12	67.9	6.44	63.8	72.0	<0.001
POW4	12	30.6	5.20	27.3	33.9	12	66.3	6.10	62.5	70.2	<0.001
POW6	12	29.3	3.55	27.1	31.6	12	55.1	4.56	52.2	58.0	<0.001

OHIP score (QoL)	Group										p-val
	Study					Control					
	N	Mean	SD	LL	UL	N	Mean	SD	LL	UL	
Post-trauma	12	49.6	2.11	48.2	50.9	12	49.8	1.90	48.6	51.0	0.763
POD2	12	43.6	4.03	41.0	46.1	12	50.2	2.04	48.9	51.5	<0.001
POW1	12	22.2	5.31	18.8	25.5	12	43.3	2.18	41.9	44.6	<0.001
POW2	12	19.0	3.54	16.7	21.3	12	37.8	2.29	36.4	39.3	<0.001
POW4	12	15.8	.87	15.2	16.3	12	33.8	2.09	32.4	35.1	<0.001
POW6	12	15.6	.51	15.3	15.9	12	32.7	1.78	31.5	33.8	<0.001

Mean difference between Study and Control Groups (independent samples T-test)

	Mean Difference	95% CI Mean Diff	
		Lower	Upper
Moed score (Hard tissue): POW4	-.167	-.414	.081
Moed score (Hard tissue): POW8	-.083	-.373	.207
Landry score (Soft tissue): POD5	-.083	-.724	.557
Landry score (Soft tissue): POD9	.000	-.505	.505
Landry score (Soft tissue): POD15	.167	-.209	.542
Landry score (Soft tissue): POD21	.000	-.244	.244
Zung score (Stress): Post-trauma	-.917	-4.272	2.439
Zung score (Stress): POD2	-10.750	-15.144	-6.356
Zung score (Stress): POW1	-26.917	-32.460	-21.374
Zung score (Stress): POW2	-36.417	-41.560	-31.273
Zung score (Stress): POW4	-35.750	-40.545	-30.955
Zung score (Stress): POW6	-25.750	-29.211	-22.289
OHIP score (QoL): Post-trauma	-.250	-1.949	1.449
OHIP score (QoL): POD2	-6.583	-9.345	-3.822
OHIP score (QoL): POW1	-21.083	-24.621	-17.546
OHIP score (QoL): POW2	-18.833	-21.358	-16.308
OHIP score (QoL): POW4	-18.000	-19.397	-16.603
OHIP score (QoL): POW6	-17.083	-18.238	-15.929

Paired Samples Statistics to compare mean values between baseline and subsequent time points

Group	Moed score (Hard tissue)	Paired Differences			p-val
		Mean	95% CI		
			CL	ULr	
Study	Pre-op - POW4	-1.833	-2.081	-1.586	<0.001
	Pre-op - POW8	-2.833	-3.081	-2.586	<0.001
Control	Pre-op – POW1	-2.000*			-
	Pre-op - POW8	-2.917	-3.100	-2.733	<0.001

* As SD is zero CI and p-value cannot be calculated

Group	Landry score (Soft tissue)	Paired Differences			p-value
		Mean	95% CI		
			LL	UL	
Study	POD5 - POD9	-.333	-.646	-.020	0.039
	POD5 - POD15	-.750	-1.145	-.355	0.002
	POD5 - POD21	-.833	-1.200	-.467	<0.001
Control	POD5 - POD9	-.250	-.537	.037	0.082
	POD5 - POD15	-.500	-.832	-.168	0.007
	POD5 - POD21	-.750	-1.229	-.271	0.005

Group	Zung score (Stress)	Paired Differences			p-val
		Mean	95% CI		
			LL	UL	
Study	Post-trauma - POD2	10.000	7.224	12.776	<0.001
	Post-trauma - POW1	24.333	19.987	28.679	<0.001
	Post-trauma - POW2	32.917	29.267	36.566	<0.001
	Post-trauma - POW4	33.833	30.160	37.507	<0.001
	Post-trauma - POW6	35.083	32.423	37.744	<0.001
Control	Post-trauma - POD2	.167	-.429	.762	0.551
	Post-trauma - POW1	-1.667	-3.987	.653	0.142
	Post-trauma - POW2	-2.583	-5.983	.816	0.123
	Post-trauma - POW4	-1.000	-4.318	2.318	0.521
	Post-trauma - POW6	10.250	7.553	12.947	<0.001

Group	OHIP score (QoL)	Paired Differences			p-val
		Mean	95% CI		
			LL	UL	
Study	Post-trauma - POD2	6.000	4.541	7.459	<0.001
	Post-trauma - POW1	27.417	24.621	30.212	<0.001
	Post-trauma - POW2	30.583	29.088	32.079	<0.001
	Post-trauma - POW4	33.833	32.329	35.338	<0.001
	Post-trauma - POW6	34.000	32.645	35.355	<0.001
Control	Post-trauma - POD2	-.333	-2.403	1.736	0.730
	Post-trauma - POW1	6.583	4.425	8.742	<0.001
	Post-trauma - POW2	12.000	9.816	14.184	<0.001
	Post-trauma - POW4	16.083	13.977	18.190	<0.001
	Post-trauma - POW6	17.167	15.160	19.173	<0.001

Non-Parametric test

S. Osteocalcin (Biochemical)	Group												p-val*
	Study						Control						
	N	Med	LL	UL	Q1	Q3	N	Med	LL	UL	Q1	Q3	
Pre-op	12	11.6	4.7	22.8	6.2	22.1	12	18.1	8.9	28.2	9.1	26.7	0.319
Post-op	12	22.6	12.5	30.0	15.0	29.0	12	33.1	14.0	48.7	14.9	44.0	0.478
p-val@	0.003						0.002						

* Mann-Whitney U Test to compare values between Study and Control Groups

@ Wilcoxon Signed Rank Test to compare values between Pre-op and Post-op time points

BOX plot

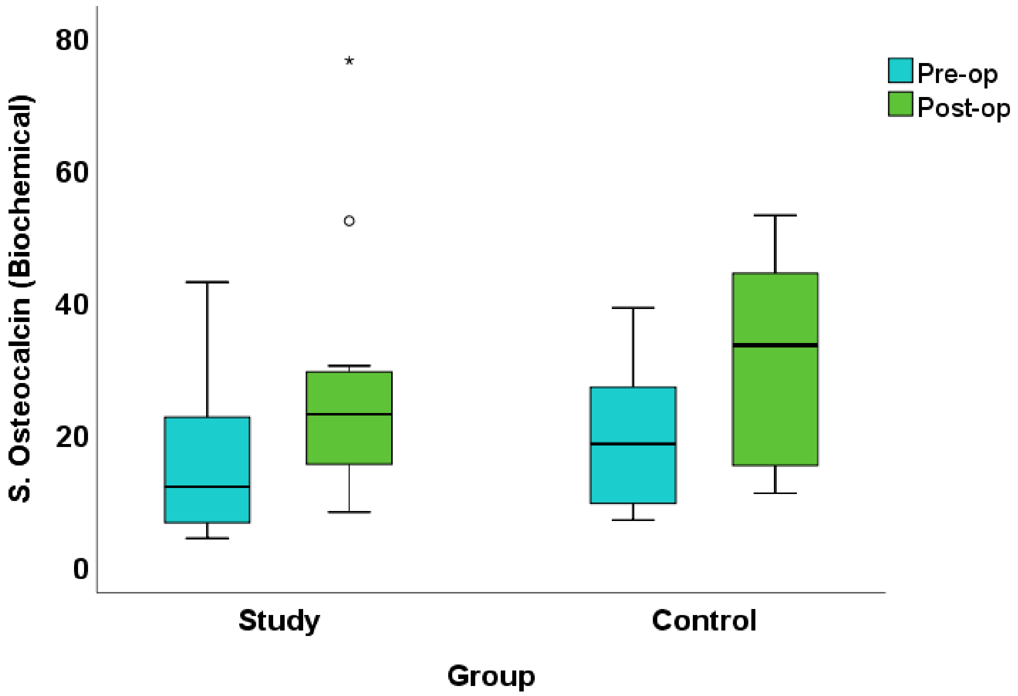

Supplement: Supplementary file 1 [file cmtr-18-00037-s001.zip › 5. Statistical workup.pdf]
