# Supplementary figures and images for: Impact of Therapeutic Alcohol Administration on Perioperative Quality of Life (QoL) and Fracture Healing in Patients with Alcohol Use Disorder Undergoing Surgery for Maxillofacial Trauma—A Randomized Pilot Trial
Source: Craniomaxillofac Trauma Reconstr. 2025 Aug 30;18(3):37. doi: 10.3390/cmtr18030037 (PMC12452396; doi:10.3390/cmtr18030037)

# CONSORT Flowchart

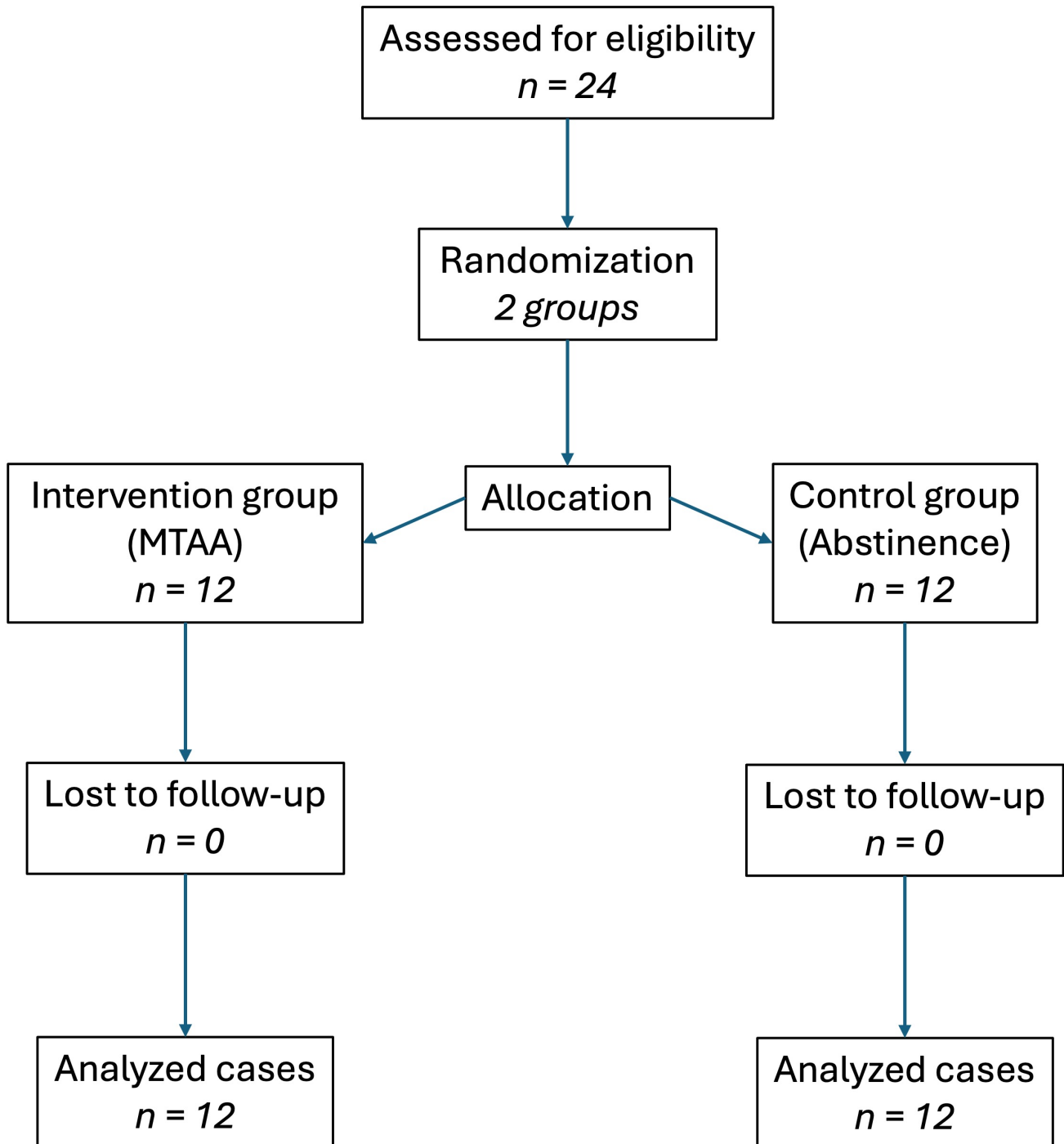

Supplement: Supplementary file 1 [file cmtr-18-00037-s001.zip › 3. Consort flowchart.pdf]
